# Exosome-Based Therapy for Skin Complications in Oncology Patients Treated with EGFR Inhibitors: A Case Report Highlighting the Need for Coordinated Dermato-Oncologic Care

**DOI:** 10.3390/ph18081090

**Published:** 2025-07-23

**Authors:** Lidia Majewska, Karolina Dorosz, Jacek Kijowski

**Affiliations:** 1 ESME Clinic, ul. Lwowska 1/U16, 30-548 Kraków, Poland; 2 Biology Division, University of Chicago, Chicago, IL 60637, USA; kdorosz@uchicago.edu; 3 Stem Cell Laboratory, Małopolskie Centrum Biotechnologii UJ, ul. Gronostajowa 7A, 30-387 Kraków, Poland; jacek.kijowski@uj.edu.pl

**Keywords:** EGFR inhibitors, dermatologic side effects, papulopustular rash, rose stem cell-derived exosomes, dermato-oncologic care, acneiform rash, anti-inflammatory treatment, oncology patients, EGFRI-induced rash, quality of life, panitumumab, innovative dermatological therapies, coordinated care, corticosteroid alternative

## Abstract

Patients undergoing epidermal growth factor receptor inhibitor (EGFRI) therapy frequently experience dermatologic side effects, notably papulopustular rash, which impacts 50–90% of recipients. This rash typically appears on the face, chest, and back within weeks of treatment, resembling acne but stemming from distinct pathophysiological mechanisms, causing significant discomfort and reduced quality of life. Prophylactic measures and symptom-based treatment are recommended, emphasizing patient education, topical agents, and systemic therapies for severe cases. A 41-year-old female with advanced colonic mucinous adenocarcinoma developed severe acneiform rash and pruritus during EGFRI therapy with panitumumab. Initial standard treatment with oral doxycycline was discontinued after two days due to severe gastrointestinal intolerance characterized by intense nausea and dyspepsia. With limited access to dermatological consultation, treatment with rose stem cell-derived exosomes (RSCEs) provided rapid symptom relief. Significant improvement was observed within 24 h, with complete resolution of pruritus and substantial reduction in inflammatory lesions within 72 h. RSCEs demonstrate anti-inflammatory effects through the modulation of pro-inflammatory cytokines including interleukin-6, interleukin-1β, and tumor necrosis factor-α, while promoting fibroblast proliferation and collagen synthesis enhancement. They may represent a possible alternative to corticosteroids, avoiding associated side effects such as skin atrophy, delayed wound healing, and local immunosuppression. This case underscores the potential of innovative treatments like RSCEs in managing EGFRI-induced skin complications when standard therapies are not tolerated, particularly in healthcare systems with limited dermato-oncological resources.

## 1. Introduction

Patients receiving epidermal growth factor receptor inhibitors (EGFRIs) commonly encounter dermatologic complications, with papulopustular rash being the most prevalent adverse effect, occurring in 50–90% of patients undergoing EGFRI therapy for malignancies such as lung, colorectal, and head and neck cancers [1,2]. This rash typically appears on the face, chest, and back within the first few weeks of treatment; although resembling acne, it stems from distinct pathophysiological mechanisms involving EGFR signaling disruption in keratinocytes, leading to impaired epidermal barrier function and inflammatory cascade activation, often accompanied by significant pain, pruritus, and discomfort [2,3].

Current management strategies emphasize early prophylactic measures, with the Multinational Association of Supportive Care in Cancer (MASCC) guidelines recommending prophylactic use of oral doxycycline at 100 mg twice daily or minocycline at 100 mg once daily for the first six weeks of EGFRI therapy, which represents the standard of care for preventing severe dermatologic toxicity [4]. Studies have demonstrated that prophylactic tetracycline-class antibiotics (doxycycline and minocycline) significantly reduce the severity of skin reactions, with evidence showing that doxycycline at 100–200 mg daily or minocycline at 100 mg daily administered from the first day of EGFR-inhibitor treatment effectively diminishes grade 2 and greater skin toxicities by more than 50% [4,5]. Notably, several agents are not recommended, including systemic tetracycline at higher doses such as 500 mg twice daily, which failed to demonstrate consistent benefit in randomized controlled trials, whereas lower-dose tetracycline at 250 mg twice daily showed efficacy for specific EGFR-TKIs like afatinib [6,7].

However, in many healthcare systems, limited access to coordinated dermato-oncological care leaves patients seeking alternative solutions independently, often resulting in treatment delays and reduced quality of life, particularly when standard therapies are not tolerated due to adverse effects [8].

Despite the established efficacy of prophylactic doxycycline therapy, a significant research gap exists in managing patients who experience gastrointestinal intolerance to standard treatments, leaving clinicians with limited therapeutic alternatives. The current literature lacks documentation of effective alternative approaches for this patient population, particularly in healthcare systems with restricted access to specialized dermato-oncological care. This represents a critical unmet clinical need, as treatment discontinuation or inadequate symptom management can lead to dose modifications or therapy interruption, potentially compromising oncological outcomes. The present case report addresses this gap by documenting the first clinical application of rose stem cell-derived exosomes (RSCEs) for EGFR inhibitor-induced skin toxicity. This novel therapeutic approach represents a paradigm shift from conventional antibiotic-based management to plant-derived exosome technology, offering a new class of anti-inflammatory agents with distinct mechanisms of action. The innovation lies not only in the therapeutic modality but also in its accessibility as an over-the-counter treatment option, potentially transforming management strategies for patients with limited access to specialized care.

RSCEs were selected as an alternative approach due to their documented anti-inflammatory properties, topical administration route avoiding gastrointestinal side effects, and over-the-counter availability when standard topical antibiotics and other anti-inflammatory agents were either unavailable or contraindicated.

## 2. Case Report

A 41-year-old female with no significant medical history was diagnosed with advanced colonic mucinous adenocarcinoma (stage IVc) with peritoneal metastases. Following left-sided hemicolectomy in December 2023, histologic examination confirmed colonic mucinous adenocarcinoma G2, KRAS, NRAS, BRAF wild-type, and microsatellite stable. In February 2024, the patient was qualified for FOLFOX + panitumumab chemotherapy based on tumor location, histopathological subtype, molecular status, and performance status (WHO: 1). The first cycle was administered with good initial tolerance.

In May 2024, an acneiform rash appeared on the patient’s face, accompanied by intense pruritus, subsequently appearing bilaterally in the hip region with generalized pruritus. Her primary care physician prescribed oral antihistamine and 1% hydrocortisone cream. Consistent with standard care protocols for EGFR-induced skin toxicity, oral doxycycline at 100 mg twice daily was initiated as recommended by current guidelines for prophylactic management. However, the patient experienced severe gastrointestinal adverse effects, including intense nausea and dyspepsia of significant severity, necessitating discontinuation after two days despite the recognized importance of completing the full course of antibiotic therapy.

This intolerance to first-line systemic therapy, combined with a six-month wait for dermatological consultation, prompted the search for alternative therapeutic approaches. Given the patient’s deteriorated psychological state, significant decline in quality of life due to persistent symptoms, associated social withdrawal, and documented intolerance to standard doxycycline therapy, we opted to initiate treatment with rose stem cell-derived exosomes (RSCEs) after obtaining detailed informed consent. The patient was thoroughly informed about the nature of the therapy, potential benefits and risks, the limited evidence base for this novel approach, and her right to discontinue treatment at any time while maintaining access to standard care options.

**Figure 1:**
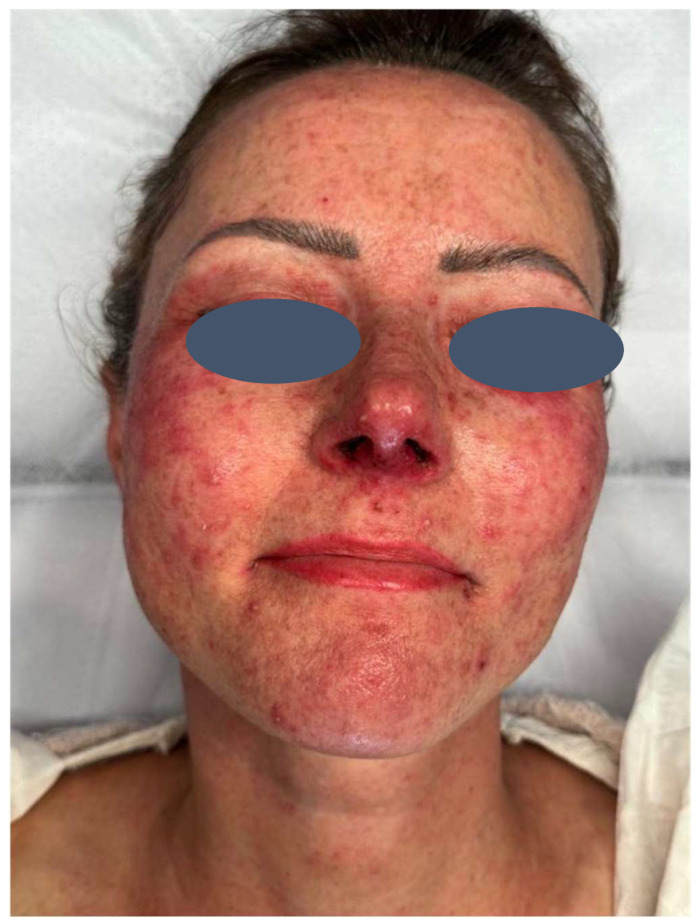
Day 0—Baseline presentation before treatment initiation. Severe acneiform rash on facial region showing multiple inflammatory papules, pustules, and erythematous lesions characteristic of EGFR inhibitor-induced skin toxicity.

**Figure 2:**
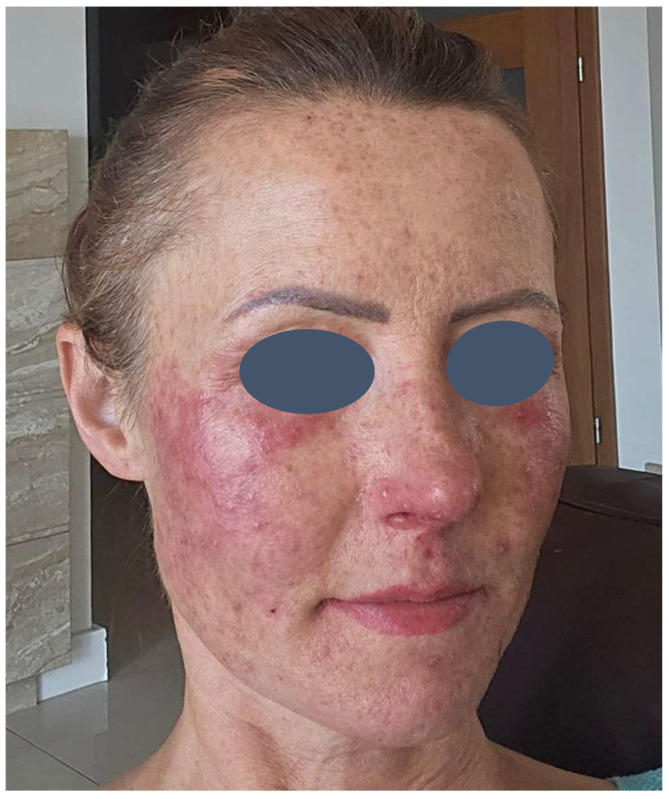
Day 1—Clinical improvement at 24 h post-treatment. Notable reduction in inflammatory lesions and erythema following initial application of rose stem cell-derived exosomes (ASCEplus/SRLV™).

**Figure 3:**
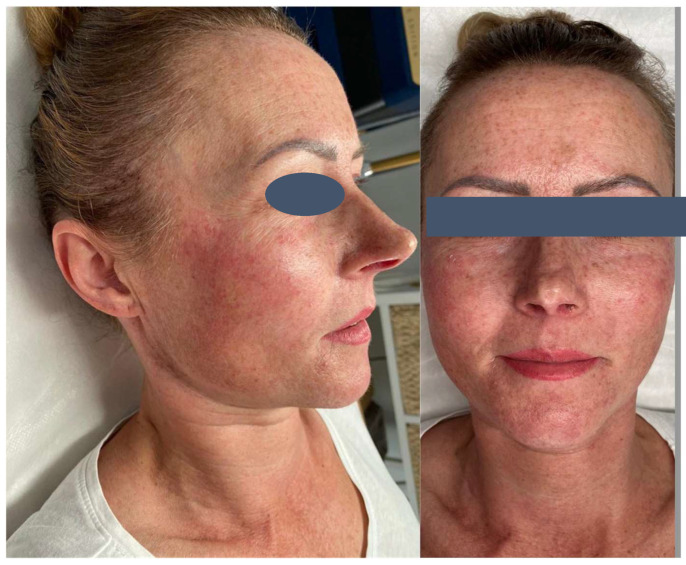
Day 3—Treatment response at 72 h. Significant improvement with near-complete resolution of inflammatory changes and restoration of normal skin appearance.

The patient presented with severe acneiform rash on her face accompanied by intense pruritus (Figure 1). Treatment was initiated with ASCEplus/SRLV™ (ExoCoBio Inc., Seoul, Republic of Korea), containing exosomes derived from Damask rose stem cells. Two layers were applied in-clinic, with remaining product provided for twice-daily home application to affected areas. At 24 h follow-up, substantial improvement in facial lesions was observed (Figure 2), with complete resolution of pruritus within one hour of initial application, demonstrating the rapid onset of the therapeutic effects characteristic of exosome-based interventions. By 72 h, a significant reduction in inflammatory changes and complete symptom resolution were documented (Figure 3).

**Figure 4:**
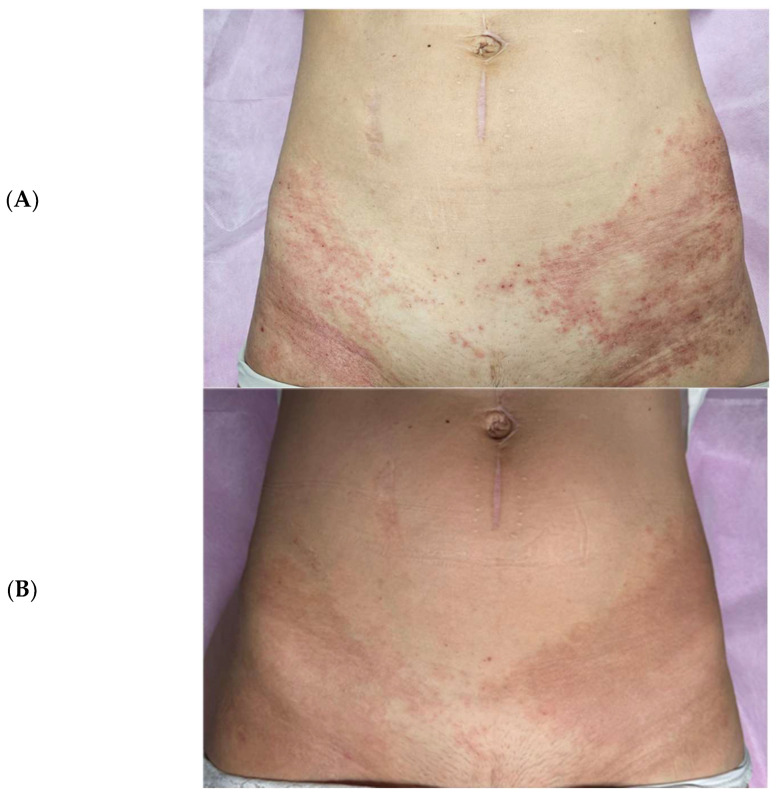
Hip region lesions before and after treatment. (**A**) Baseline appearance showing erythematous lesions and inflammation in the hip region before ExoBalm™ application. (**B**) Complete resolution of rash, erythema, and irritation after 72 h of twice-daily ExoBalm™ treatment, demonstrating the efficacy of RSCE-based therapy in different anatomical locations.

Subsequently, treatment was extended to hip region lesions using ExoBalm™ (ExoCoBio Inc., Seoul, Republic of Korea), a formulation containing RSCEs with tranexamic acid, madecassoside, D-panthenol, and niacinamide. Complete resolution of rash, erythema, and irritation was achieved within three days of twice-daily application (Figure 4), with the patient reporting enhanced comfort and visible improvements in skin texture compared to the prolonged inflammatory state experienced with standard treatments.

## 3. Discussion

### 3.1. Pathophysiology of EGFR Inhibitor-Induced Skin Toxicity

EGFR inhibitors cause characteristic dermatologic changes through a blockade of epidermal growth factor receptor signaling, which plays crucial roles in keratinocyte proliferation, differentiation, and survival [1,2]. This disruption leads to impaired epidermal barrier function and increased transepidermal water loss, disrupted keratinization processes, and the activation of inflammatory cascades involving pro-inflammatory cytokines including interleukin-1α, interleukin-1β, and tumor necrosis factor-α [2,3]. Secondary bacterial colonization, particularly with Staphylococcus aureus, and altered hair follicle function contribute to the acneiform appearance. These mechanisms differ fundamentally from acne vulgaris pathogenesis, explaining why conventional acne treatments are often ineffective and why retinoids may exacerbate EGFR-induced skin toxicity [3].

### 3.2. Clinical Significance of Rapid Therapeutic Response

The rapid therapeutic response observed in this single case, with symptom relief occurring within hours and significant improvement within 24 h, suggests that RSCEs could potentially represent a therapeutic option for oncology patients. EGFR inhibitor-induced skin toxicity significantly impacts patient quality of life, with studies demonstrating that severe dermatologic reactions can lead to treatment modifications in up to 76% of cases, potentially compromising oncological outcomes [9]. The ability to provide immediate symptom relief while maintaining the patient’s cancer treatment regimen represents a critical clinical advantage. The psychological burden of visible skin lesions, particularly facial involvement, cannot be understated in cancer patients who are already coping with significant emotional stress. The rapid resolution of symptoms observed with RSCE therapy may help prevent the social withdrawal and depression commonly associated with severe dermatologic toxicity, thereby maintaining patient adherence to life-saving cancer treatments.

### 3.3. Healthcare System Implications and Accessibility

The over-the-counter availability of RSCE-based products addresses a critical gap in healthcare delivery, particularly relevant in systems with limited dermato-oncological resources. Our patient faced a six-month wait for dermatological consultation, a delay that is unfortunately common in many healthcare systems worldwide. The accessibility of effective self-administered treatment options can bridge this care gap, providing patients with immediate therapeutic alternatives while awaiting specialist consultation. This accessibility becomes particularly important when considering the global burden of cancer care and the increasing use of EGFR inhibitors across diverse healthcare settings. The ability to manage severe skin toxicity without requiring immediate specialist intervention may enable the broader implementation of life-saving cancer therapies in resource-limited settings.

### 3.4. Economic and Practical Considerations

The cost-effectiveness implications of RSCE therapy warrant consideration. While the initial cost of over-the-counter exosome products may appear higher than generic doxycycline, the comprehensive economic analysis must include factors such as healthcare utilization for side effect management, productivity losses due to symptom burden, and potential costs associated with cancer treatment modifications. The rapid symptom resolution observed may reduce the need for multiple healthcare visits, specialist consultations, and time away from work or normal activities. Furthermore, the topical administration route eliminates concerns about drug interactions and gastrointestinal complications that may necessitate additional medical management, potentially reducing overall healthcare costs and improving patient convenience.

### 3.5. Standard Treatment Approaches and Limitations

Current evidence-based management of EGFR inhibitor-induced papulopustular rash primarily focuses on prophylactic antibiotic therapy, with the MASCC guidelines establishing doxycycline at 100 mg twice daily or minocycline at 100 mg once daily as the standard prophylactic regimen for the first six weeks of therapy [4]. Clinical studies have demonstrated that prophylactic treatment with tetracycline-class therapeutics (doxycycline and minocycline) significantly reduces the severity of skin reactions across various indications, including metastatic colorectal cancer and non-small-cell lung cancer [4,5]. The anti-inflammatory effects of these antibiotics, rather than their antimicrobial properties, underlie their efficacy in managing EGFR-related skin toxicity through the inhibition of mitogen-induced lymphocyte proliferation, the suppression of neutrophil and lymphocyte chemotaxis, the upregulation of anti-inflammatory cytokine interleukin-10, and the downregulation of interleukin-6 [6,7].

However, gastrointestinal intolerance represents a significant limitation of doxycycline therapy, with common adverse effects including nausea, dyspepsia, and abdominal cramping that may necessitate treatment discontinuation in susceptible patients [7]. Doxycycline appears to have a more favorable safety profile, especially in patients with renal dysfunction, whereas minocycline is less photosensitizing, thus preferable in geographic or seasonal locations with a high ultraviolet index [4]. For patients requiring systemic corticosteroids due to severe grade 3 reactions, treatment typically involves methylprednisolone dose packs or prednisone at 0.5 mg per kilogram for seven days, though prolonged corticosteroid use carries substantial risks [4].

### 3.6. Mechanisms of Action of Rose Stem Cell-Derived Exosomes

It should be emphasized that the specific mechanisms of RSCEs in EGFR inhibitor-induced skin toxicity remain theoretical and largely unknown. The mechanistic explanations provided are based on the general plant-derived exosome literature and require specific investigation in this clinical context.

RSCEs represent a novel therapeutic approach that exerts anti-inflammatory effects through multiple sophisticated mechanisms distinct from conventional treatments [10,11,12]. Recent research has demonstrated that plant-derived exosome-like nanoparticles, including those derived from rose stem cells, contain specific bioactive molecules including microRNAs, proteins, and lipids that facilitate intercellular communication and modulate inflammatory responses [12]. RSCEs specifically downregulate pro-inflammatory cytokine expression including interleukin-6, interleukin-1β, and tumor necrosis factor-α, while upregulating anti-inflammatory mediators such as interleukin-10 through mechanisms involving nuclear factor-κB pathway modulation and inflammasome inhibition [11].

The anti-inflammatory properties of rose-derived compounds have been extensively documented, with rose petal extracts containing flavonoids, anthocyanins, and polyphenols demonstrating significant reduction in solar ultraviolet-induced expression of cyclooxygenase-2 and the inhibition of multiple inflammatory cytokines through mitogen-activated protein kinase pathway inactivation [11]. RSCEs facilitate wound healing through the enhancement of fibroblast proliferation and migration, the stimulation of collagen synthesis, and the promotion of angiogenesis via growth factor delivery including vascular endothelial growth factor and fibroblast growth factor [11]. The regenerative mechanisms involve the transfer of regulatory microRNAs including miR-146a and miR-155 that modulate inflammatory signaling cascades, while heat-shock proteins support cellular repair processes [11].

Unlike traditional anti-inflammatory approaches, RSCEs may demonstrate potentially favorable safety profiles by avoiding the adverse effects associated with prolonged corticosteroid use [13,14,15]. Topical corticosteroids, while effective for acute inflammatory control, are associated with significant long-term complications including skin atrophy, striae formation, telangiectasia, delayed wound healing, and local immunosuppression [13,14]. Corticosteroids impair wound healing through the inhibition of fibroblast proliferation, reduced collagen synthesis, compromised angiogenesis, and the suppression of epithelialization, with these effects being particularly pronounced with high-potency formulations or prolonged application [13,14]. The anti-inflammatory effects of corticosteroids result from their ability to reduce collagen and ground substance formation, compromise vascular connective tissue support, and delay granulation tissue formation, ultimately leading to compromised skin integrity and increased susceptibility to injury [13,14].

### 3.7. Clinical Implications and Therapeutic Potential

The rapid therapeutic response observed in this case, with symptom relief occurring within hours and significant improvement within 24 h, suggests that RSCEs may represent a valuable therapeutic option for patients experiencing intolerance to standard treatments [9]. The mechanism underlying this rapid response likely involves the immediate anti-inflammatory effects of exosomal cargo delivery, including microRNAs that can quickly modulate gene expression and inflammatory mediator production at the cellular level [16,17]. Plant-derived exosomes have demonstrated the ability to be taken up by mammalian cells through specific endocytosis mechanisms, enabling the targeted delivery of therapeutic molecules while minimizing off-target effects [18,19,20,21,22,23].

The over-the-counter availability of RSCE-based products adds practical value, particularly in healthcare systems with limited dermato-oncological resources where access to specialized care may be delayed [9]. This accessibility becomes particularly important given that dermatologic toxicities can lead to dose modifications or treatment discontinuation in up to 76% of providers when severe reactions occur, potentially compromising oncologic outcomes [9]. The ability to provide effective symptomatic relief while maintaining cancer treatment continuity represents a significant clinical advantage [21].

Recent clinical evidence supports the therapeutic potential of exosome-based treatments across various dermatologic conditions [24,25]. Studies have demonstrated that exosomes derived from various sources can effectively manage inflammatory skin conditions, promote wound healing, and reduce scarring through the modulation of macrophage polarization, the enhancement of cellular proliferation, and the restoration of tissue homeostasis [19,20,23]. The immunomodulatory properties of exosomes, combined with their natural origin and biocompatibility, position them as promising alternatives to conventional therapies, particularly in cases where traditional treatments are contraindicated or not tolerated [23,24].

### 3.8. Study Limitations and Future Directions

This case report has several important limitations that must be acknowledged. The single-patient observation, absence of a control group, and lack of objective inflammatory biomarker assessment limit the generalizability of findings. The rapid response observed may not be reproducible in all patients, and individual factors including genetic polymorphisms, concurrent medications, and disease severity may influence therapeutic outcomes. While the rapid objective improvement in visible lesions suggests therapeutic efficacy beyond the placebo effect, the subjective symptom relief, particularly pruritus resolution, may have been influenced by a placebo response, which cannot be excluded in this uncontrolled single-case observation.

Additionally, the mechanism of action of RSCEs in EGFR-induced skin toxicity remains incompletely understood, warranting further investigation into the specific molecular pathways involved.

Future research should prioritize controlled clinical studies comparing RSCEs with standard therapies, including head-to-head comparisons with tetracycline antibiotics in patients able to tolerate such treatments. Biomarker analyses measuring inflammatory cytokine levels, skin barrier function parameters, and tissue regeneration markers would provide objective evidence of therapeutic efficacy. Longer-term safety assessments are essential to establish the safety profile of repeated RSCE applications, while dose–response studies could optimize treatment protocols. An investigation of combination therapies incorporating RSCEs with conventional treatments may reveal synergistic effects that could enhance therapeutic outcomes while minimizing adverse effects [24,25].

The development of standardized protocols for RSCE production, quality control measures, and clinical application guidelines will be crucial for translation into routine clinical practice. Additionally, cost-effectiveness analyses comparing exosome-based treatments with current standard care approaches will inform healthcare policy decisions and reimbursement considerations.

The single-patient design of this case report, while appropriate for documenting novel therapeutic approaches and rare clinical scenarios, inherently limits generalizability. However, case reports serve a crucial role in clinical medicine by documenting innovative treatments, rare adverse events, and therapeutic responses in specific patient populations. This case represents the first documentation of RSCE therapy for EGFR inhibitor-induced skin toxicity, establishing proof of concept for future larger studies. The rapid response observed provides compelling evidence that justifies progression to controlled clinical trials, which would not have been possible without this initial case documentation.

## 4. Conclusions

This case demonstrates the potential efficacy of rose stem cell-derived exosomes in managing EGFR inhibitor-induced skin toxicity when standard treatments are not tolerated due to adverse effects. The rapid symptom relief, excellent tolerability profile, and absence of the adverse effects associated with corticosteroids suggest that RSCEs may represent a valuable addition to the therapeutic armamentarium for managing oncologic dermatologic complications. The anti-inflammatory mechanisms involving cytokine modulation, combined with regenerative properties promoting tissue repair, position RSCEs as a promising alternative, particularly for patients experiencing gastrointestinal intolerance to standard doxycycline therapy.

However, it is important to emphasize that no causality can be definitively confirmed based on this single-case observation, and randomized controlled trials are essential to establish efficacy and safety before any definitive therapeutic recommendations can be made.

The experimental nature of this treatment approach necessitates cautious interpretation of these findings pending larger controlled clinical studies. The success observed in this case warrants further investigation through randomized controlled trials to establish efficacy and safety profiles, determine optimal dosing regimens, and identify patient populations most likely to benefit from exosome-based therapy. Until such evidence becomes available, RSCE treatment should be considered as an investigational approach for carefully selected patients who have experienced treatment failure or intolerance with standard therapies, always with appropriate informed consent and continued monitoring for both therapeutic response and potential adverse effects.

## Data Availability

The datasets used and analyzed during the current study are available from the corresponding author upon reasonable request, subject to patient privacy considerations.
